# Monitoring of Interfacial Debonding of Concrete Filled Pultrusion-GFRP Tubular Column Based on Piezoelectric Smart Aggregate and Wavelet Analysis

**DOI:** 10.3390/s20072149

**Published:** 2020-04-10

**Authors:** Wenwei Yang, Xia Yang, Shuntao Li

**Affiliations:** School of Civil and Hydraulic Engineering, Ningxia University, Yinchuan 750021, China; yww@nxu.edu.cn (W.Y.); lishuntaoqqq@live.com (S.L.)

**Keywords:** concrete filled pultrusion-GFRP tubular column (CFGC), interfacial debonding, smart aggregate (SA), Lead Zirconate Titanate (PZT), health monitoring, wavelet packet energy

## Abstract

The concrete filled pultrusion-GFRP (Glass Fiber Reinforced Polymer) tubular column (CFGC) is popular in hydraulic structures or regions with poor environmental conditions due to its excellent corrosion resistance. Considering the influence of concrete hydration heat, shrinkage, and creep, debonding may occur in the interface between the GFRP tube and the concrete, which will greatly reduce the cooperation of the GFRP tube and concrete, and will weaken the mechanical property of CFGC. This paper introduces an active monitoring method based on the piezoelectric transducer. In the active sensing approach, the smart aggregate (SA) embedded in the concrete acted as a driver to transmit a modulated stress wave, and the PZT (Lead Zirconate Titanate) patches attached on the outer surface of CFGC serve as sensors to receive signals and transfer them to the computer for saving. Two groups of experiments were designed with the different debonding areas and thicknesses. The artificial damage of CFGC was identified and located by comparing the value of the delay under pulse excitation and the difference of wavelet-based energy under sweep excitation, and the damage indexes were defined based on the wavelet packet energy to quantify the level of the interface damage. The results showed that the debonding damage area of CFGC can be identified effectively through the active monitoring method, and the damage index can accurately reflect the damage level of the interface of GFRP tube and concrete. Therefore, this method can be used to identify and evaluate the interface debonding of CFGC in real time. In addition, if the method can be combined with remote sensing technology, it can be used as a real-time remote sensing monitoring technology to provide a solution for interface health monitoring of CFGC.

## 1. Introduction

Glass fiber reinforced polymer (GFRP) is a kind of composite material which is made of resin and glass fiber, with resin as a matrix and glass fiber as a reinforcing material [1,2,3]. It has the advantages of high tensile strength, light weight, good durability, good elastic deformation, high electrical insulation performance, good heat resistance, and a thermal expansion coefficient close to concrete. In recent years, it has become one of the research hotspots in the field of civil engineering at home and abroad [4,5,6]. GFRP pipes are divided into pultruded GFRP pipes and wound GFRP pipes according to their different forming methods. Compared with the wound GFRP pipe, the pultruded GFRP pipe adopts one-shot molding technology, and its overall mechanical performance is good, while no lamellar tearing occurs during the force. At the same time, during the process of the column being poured, the pultruded GFRP pipe can replace the formwork, save costs, speed up the construction speed, and facilitate on-site construction [7]. 

The CFGC (concrete filled pultrusion-GFRP tubular column) combines the corrosion resistance of the GFRP tube and the compressive capacity of the concrete, and it exerts the combined advantages of the two, the mechanical properties are superior. The CFGC is similar to the concrete-filled steel tube (CFST) composite structure, but it overcomes the disadvantage that the CFST column is easy to be corroded. Therefore, it can be used as an important vertical or axial force member in large bridges and super high-rise buildings to replace the CFST column. In the construction process, due to the uneven temperature field caused by the hydration heat during concrete pouring [8] and the shrinkage and creep in the conservation process of CFGC, the bonding interface between the inner of GFRP tube and the concrete will be peeled and damaged. Studies have shown that the mechanical properties of composite structures with interfacial debonding are weakened [9,10,11,12,13]. Therefore, the research on the interface health monitoring of buildings like this new composite structure also shows a very broad application prospect.

Nowadays, non-destructive testing (NDT) techniques, including electromagnetic waves (EW) [14,15], X-ray diffraction [16], acoustic emission [17], ultrasonic guided waves [18,19,20], infrared thermal imaging [21], fiber optic sensors [22], and radar/microwave [23,24], are effective in structural damage detection. Although these methods can reflect the local damage state of the structure, they require complex and expensive instrument and professional interpretation, which makes it difficult to carry out real-time and on-line structure health monitoring [25].

As a new technical material, intelligent material has a broad development prospect in the fields of military, medicine, architecture, textile and clothing. Its application confers a self-diagnosis function on the structure. PZT (Lead Zirconate Titanate) is one of the most widely used intelligent materials at present. Due to its fast response, wide frequency response range, easy tailoring, and low price, it has great application potential in structural health monitoring [26,27,28,29,30,31,32]. The method of using PZT for transducers is becoming popular in the field of structural interface monitoring, including the wave propagation approach and the electromechanical impedance approach [33,34,35,36,37,38,39,40,41,42]. 

In recent years, PZT transducers were used to detect damage of concrete structures with the wave propagation method. Song (2005–2008) [43,44,45,46,47,48] proposed the concept of “smart aggregate (SA)” based on piezoelectric transducers, and by embedding the molded piezoelectric function elements into the concrete, the crack generation and development process of typical reinforced concrete members under different damage conditions are effectively monitored. Yan (2011) [49] successfully detected the simulated debonding region of CFRP confined concrete column by using the pre-embedded SA and the PZT patches bonded in the surface of CFRP, and excited by the sweep and then analyzed by Fourier spectrum. Xu (2013) [50] proposed an active interface condition monitoring approach for concrete-filled steel tubes by using PZT patches bonded on the surface of the steel tube and SAs embedded in concrete, and the artificial debonding area was detected successfully. Feng (2017) [51] used wave propagation method based on piezoelectric transducers to monitor the grouting progress of CFST columns. The test results showed that the amplitude of signal changed significantly before and after grouting, and this method can be used to achieve real-time monitoring of the grouting progress of CFST columns. The piezoelectric transducer-based active sensing method combined with wavelet packet analysis was used by Yang W (2018) [52] to monitor the soil compactness, the results showed that the method can effectively identify the soil compaction degree. Zhang (2018) [53,54] detected the damage and moisture of timber by using PZT transducer-based active sensing, the results showed that the active monitoring method based on piezoelectric transducer can better identify the damage level and the moisture change of wood.

From the above analysis, one can see that many scholars have researched the damage identification and monitoring of traditional structures based on PZT transducers, but there is little research on the damage monitoring of the new composite structures such as CFGC.

This paper presented an exploratory study of using a piezoceramic-based active sensing approach coupled with wavelet packet analysis to quantitatively monitor the interface debonding damage of CFGC in real time, and to determine the location and the level of damage. The pulsed signals and the sweep signals were used to excite the SAs in CFGC. The simulated debonding area was detected successfully by comparing the delay of the measured signal under pulse excitation and the difference of the wavelet packet energy under sweep excitation. Meanwhile, the damage indexes based on the wavelet packet energy were defined, and the results showed that the damage index is sensitive enough to the level of debonding damage in CFGC.

## 2. Rationale

### 2.1. Wave Propagation Analysis Based on Piezoelectric Transducers

**Figure 1:**
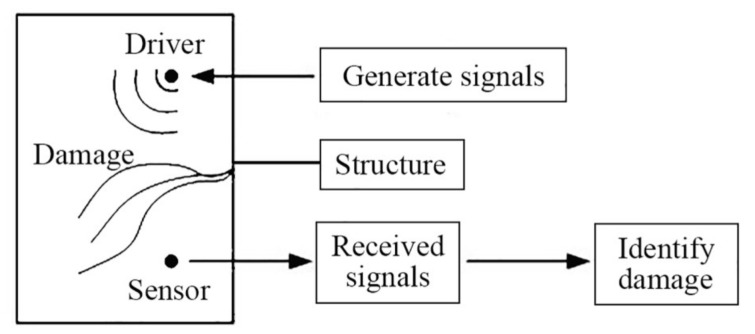
Active piezoelectric transducer detection system based on wave analysis.

This paper used the wave propagation analysis method to identify the interface damage of CFGC. The basic principle of the wave analysis method is to attach the piezoelectric transducers to the surface or the inside of the structure to form an intelligent detection system based on the piezoelectric transducer [55]. In this paper, the multi-function piezoelectric signal detection and analysis system was used to transmit and collect signals. Firstly, an analog electrical signal is used to stimulate the SA which buried in the CFGC, and the SA will convert the electrical signal into a high-frequency stress wave, and it is propagated through concrete and GFRP tube to the PZT patches bonded on the surface of CFGC. At the moment, the PZT patches will receive the high-frequency stress wave and convert it into electrical signals and transmit them to the computer through the data acquisition system. This process applies the positive and negative piezoelectric effect of piezoelectric transducers. The occurrence of damage may cause the attenuation of the signal amplitude and the wavelet packet energy, the change of the signal mode and the arrival time delay of wave, etc., therefore, the location and the level of structural damage could be identified by the difference of the acquired signals from a group of four PZT patches. The basic composition of the piezoelectric active detection system based on the wave propagation analysis method is shown in Figure 1. 

### 2.2. Wavelet Packet Energy

**Figure 2:**
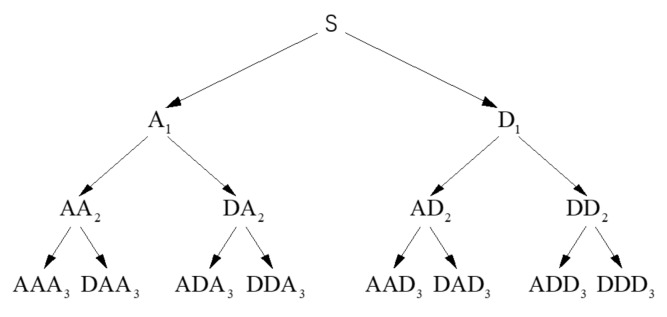
Wavelet packet decomposition at level 3.

In recent years, multifarious signal processing approaches have been applied to analyze stress wave information [56,57]. The popular approaches include wavelet/wavelet packet analysis, Fourier transform analysis, inverse mapping theory, neural network theory, statistical analysis, and reconstitution theory [58,59]. Among these methods, wavelet packet analysis has shown great potential in structural damage evaluation [60,61]. In the wavelet packet decomposition process, the original signal can be decomposed into narrow frequency bands over a relatively short time window. and can adaptively select the corresponding frequency band according to the characteristics of the signal to be analyzed. This kind of decomposition has no redundancy and no omission, which makes it better to perform local time-frequency analysis for signal. Figure 2 illustrates the wavelet decomposition at level 3. The wavelet packet energy has an equivalent relationship with the energy of original monitoring signal. Therefore, the wavelet packet energy can be used to represent the total energy of the original monitoring signal, and damage of the structure is identified by comparing the difference of the wavelet packet energy of each monitoring signal.

### 2.3. Definition of Damage Index Based on Wavelet Packet Energy

Recently, in terms of structural health monitoring, in order to better characterize the damage degree of civil engineering structures, a variety of damage indexes have been proposed by many scholars, including the root mean square index (RMSD) proposed by Giurgiutiu and Rogers (2000) [62], which is a suitable damage index that can better distinguish the health signals and the injury signals of structures. Therefore, the damage index DI can be obtained by calculating the RMSD of energy vector in the health state and the damage state of structures. The energy vector of the healthy state is denoted by *E_h_* = [*E_h,i_*], and the energy vector of the damage state is represented by *E_k_* = [*E_k,i_*]. For this, the expression is as follows:

(1) $$DI=\sqrt{\frac{\sum_{i=1}^{2^{n}}{\left(E_{k,i}-E_{h,i}\right)}^{2}}{\sum_{i=1}^{2^{n}}E_{h,i}^{2}}}\begin{array}{cc} & \left(i,=,1,,,2,,,\;,\ldots ,,,2^{N}\right) \end{array}$$

## 3. Test Introduction

### 3.1. Specimen Design

**Figure 3:**
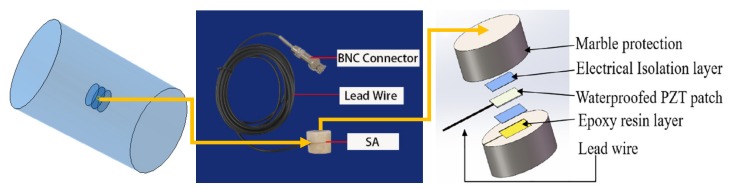
The position of SA embedded in concrete and the structural composition of SA.

**Figure 4:**
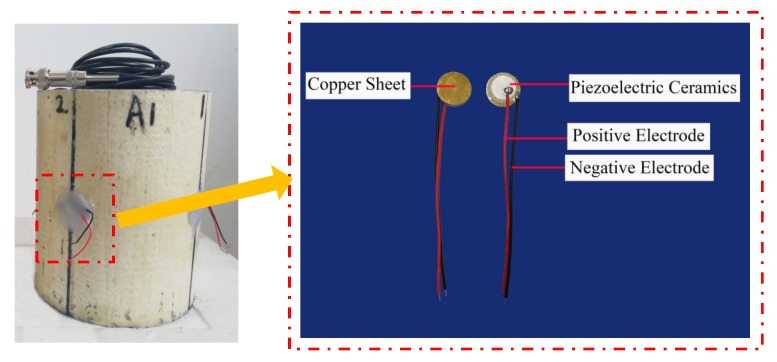
PZT patches pasted on the column.

Two groups of specimens were designed in this test, both of which were CFGCs with a height of 200 mm and a section diameter of 120 mm, and they were numbered A1 and A2. For the specimens, GFRP tube was prefabricated pultruded GFRP tube in factory, the thickness was 4 mm, the inner diameter was 120 mm. The concrete grade was C30, which is configured according to the mixing ratio of ordinary concrete, the ordinary Portland cement with 42.5R produced by Saima in Ningxia was adopted, the coarse aggregate used fine graded gravel with a particle size of not more than 10 mm, and the fine aggregate adopted ordinary medium sand. SAs were embedded in the interior of specimen A1 and A2 as shown in Figure 3, and they were used as drivers to emit signals. The two SAs embedded in specimen A1 and A2 were labeled as SA1 and SA2 respectively. As for every specimen, four PZT patches were attached to the 1/2 height of the column as sensors to receive signals from SA, they were pasted with AB glue as shown in Figure 4. In addition, we can get that the SA was composed by a waterproofed PZT patch with an electric connection wire, an electrical isolation layer covering the PZT patch, an epoxy resin bonding layer, and two mated marble pieces for protection [52] from the Figure 3. 

### 3.2. Design of Interfacial Debonding Damage 

**Figure 5:**
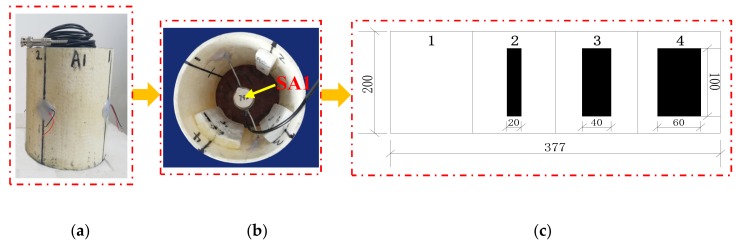
The arrangement of SA1 and the damage position of specimen A1. (**a**) Specimen A1; (**b**) Sectional view of A1; (**c**) Unfolded view of specimen A1 along the perimeter of the section.

**Figure 6:**
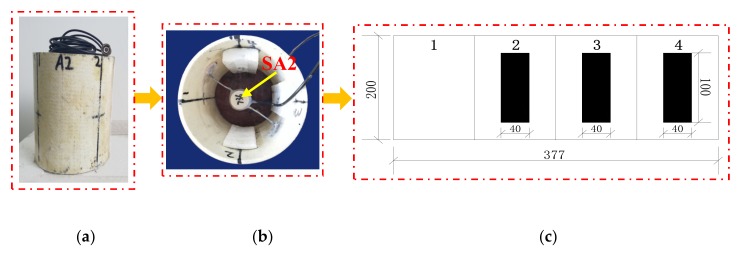
The arrangement of SA2 and the damage position of specimen A2. (**a**) Specimen A2; (**b**) Sectional view of A2; (**c**) Unfolded view of specimen A2 along the perimeter of the section.

As shown in Figure 5, the specimen A1 was divided into four equal parts along the circumference of a section, which were numbered 1, 2, 3, and 4, and then the artificial interface damage with different debonding area was produced in the three regions numbered 2, 3, and 4, respectively. Region 1 of specimen A1 was used as health conditions for reference. The simulation of damage was achieved by attaching a polystyrene foam board to the inner of GFRP tube, and they were pasted with epoxy resin binder. The damage sizes of regions numbered 2, 3, and 4 for specimen A1 were 100 × 20 × 10 mm, 100 × 40 × 10 mm, and 100 × 60 × 10 mm, respectively. As shown in Figure 6, specimen A2 had the same damage location and the damage had been made in the same way as specimen A1. The only difference was that the interface damage of specimen A2 was designed with different debonding thicknesses. So, the damage sizes of regions numbered 2, 3, and 4 for specimen A2 were 100 × 40 × 5 mm, 100 × 40 × 10 mm, and 100 × 40 × 15 mm. Region 1 of specimen A2 was also used as health conditions for reference.

### 3.3. Data Collection System

**Figure 7:**
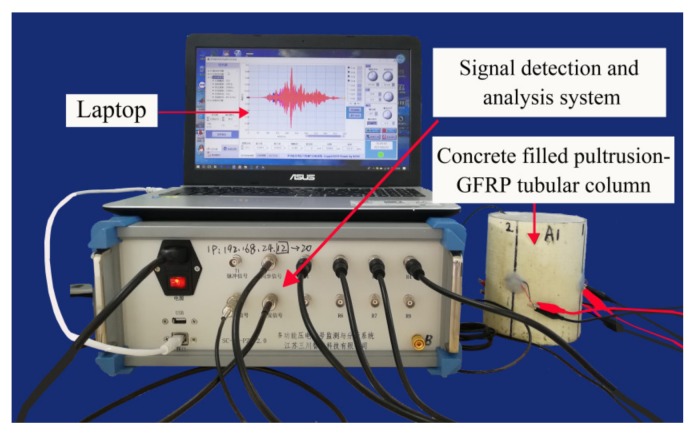
Composition of active detection system based on piezoelectric transducers.

As shown in Figure 7, the active detection system based on PZT transducers consists of the computer equipped with SCHYPZTV3 software (CES), the multi-function piezoelectric signal detection and analysis system (MPDAS) produced by Sanchuan Intelligent Technology Co., Ltd from China, and the CFGC columns under test. In the test, the CES was used to send instructions to MPDAS and collect monitoring signals, and the MPDAS was responsible for modulating and transmitting signals. The MPDAS integrates signal generator and oscilloscope, and real-time filtering can be performed in the process of data acquisition. In addition, this system can generate a variety of excitation signals, including sweep, pulse and other arbitrary signals. In this paper, authors mainly used sweep and pulse as excitation signals. Sweep is essentially a sine wave with continuously changing frequency, it can perform a linear dynamic frequency sweep for the structure. The characteristics of sweep excitation are simple and fast, and it can conveniently measure the frequency and dynamic characteristics of the system. The pulse is a discrete signal, and compared with the sweep, the waveform of pulse is discontinuous on the time axis and it is characterized by sudden changes in the moment, extremely short action time, and large instantaneous power.

In order to control the input, before the test, we have collected signals used the PZT patches with the system didn’t transmit any signal, and we obtained the environmental noise floor and recorded it. Then we excited the SA and received the signals by the PZT patches, and found that the waveforms, amplitudes, and powers of the signals collected by the system were significantly different with and without the input, from this we can determine that the signal we collected is the signal we input. Subsequently, we have performed a frequency domain analysis for the noise floor to determine its frequency range, and we can filter it by a band-pass filter to reduce its interference for the input signal. At the same time, our measurement for the specimens was not a one-time measurement, and the input range of the signal can be determined by the preliminary predictive measurement and a suitable range was selected to ensure that the acquired signal is not distorted. In addition, we can determine the effective signals were collected rather than noise by observing the correlation of the frequency between the detection signal and the input signal. Noise frequency is not related to the frequency of the input signal, and the correlation of noise and time is also very poor.

## 4. Analysis of Experimental Process and Results

### 4.1. Experimental Procedure

This test was carried out in the form of one-excitation with four-receipts by using active detection. In order to remove the influence of noise, real-time filtering was performed in the whole process of the test. At the same time, to eliminate signal distortion caused by accidental errors, the data was not saved until the wave form was stable by multiple excitations.

For specimen A1, the signal was transmitted by SA1 buried in the concrete, and the four PZT patches were applied for receiving signals. Firstly, sweep signal was used for excitation. In the process of wave propagation, when the frequency of wave is low, the wavelength is long and it may be larger than the average particle size of the concrete aggregate, in this case, the wave may bypass the concrete aggregate and the artificial damage areas so that the damage can’t be detected [30].Therefore, we need to carry out several pre-tests before the formal test to determine the range of sweep signals, and it was 50 kHz–350 kHz, and the amplitude adopted 10 V. In order to ensure the waveform was smooth, the step frequency was 0.6 kHz, the step was 1 ms, and the duration of sweep was 501 ms. Then, the pulse signal was used to excite SA1 with an amplitude of 100 V and a duration of 40 μs. The parameters of signal acquisition and filtering are given in Table 1.

For specimen A2, the signal was transmitted by SA2 buried in the concrete, and the four PZT patches attached to the pultruded GFRP tube were applied for receiving signals. Similarly, the sweep signal and pulse signal were used for excitation. Due to some accidental errors in the production process of specimens and SA, the slight difference in the performance of PZT patches, and the variation of the damage areas, the triggering and acquisition parameters of specimen A2 were different from specimen A1. Through multiple pre-tests, the selected range of sweep signal was 30–230 kHz, the amplitude was 10 V, the step frequency was 0.4 kHz, the step was 1ms, and the sweep duration was 501 ms. The amplitude of pulse was 100 V and the duration was 40 μs. Parameters of signal acquisition and filtering of the specimen A2 are given in Table 2.

### 4.2. Analysis of Test Results

#### 4.2.1. Test of Different Debonding Area Identification

For specimen A1, sweep and pulse signals were used for excitation, respectively, while the wavelet packet energy with db2 and the wavelet-based DI in different debonding area was calculated. At the same time, the delays based on pulse were extracted. The result parameters are listed in Table 3.

**Figure 8:**
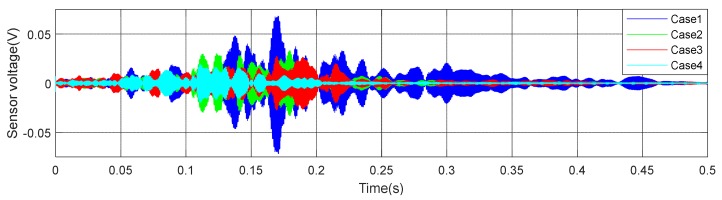
Waveform of specimen A1 under sweep excitation.

**Figure 9:**
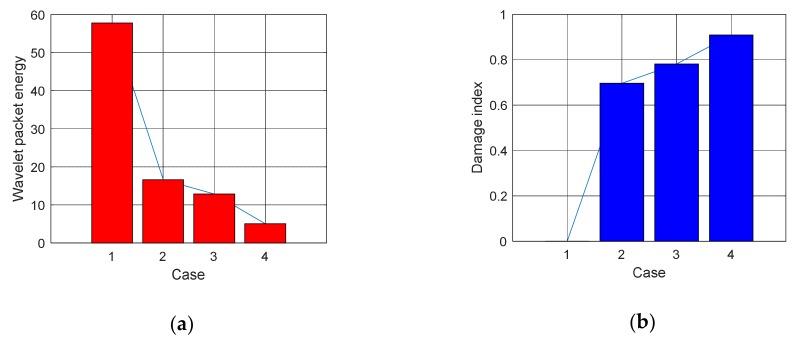
Wavelet packet energy and damage index under four cases of specimen A1. (**a**) Wavelet packet energy, (**b**) Damage index DI.

**Figure 10:**
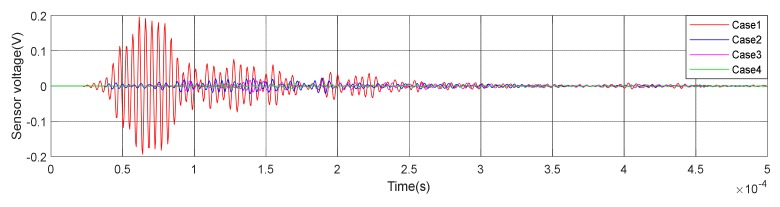
Waveform of specimen A1 under pulse excitation.

**Figure 11:**
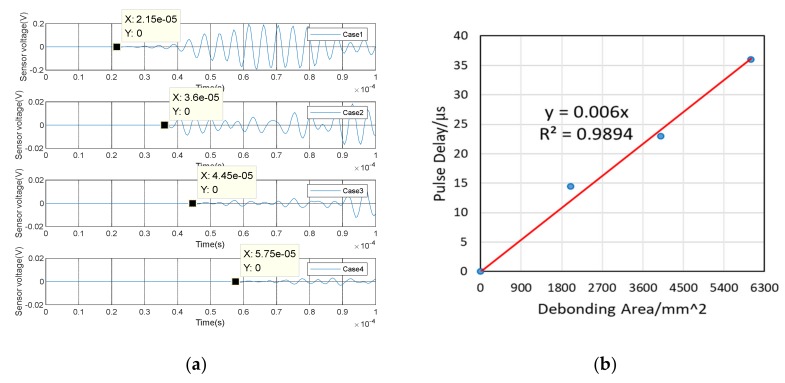
Test result of specimen A1 under pulse excitation. (**a**) Arrival time of pulse wave; (**b**) Relationship between debonding area and pulse delay.

(1) Time Domain Analysis of Sweep Signal
  The time domain of signal under the excitation of sweep was shown in Figure 8 of specimen A1. It can be seen from the Figure 8 that the amplitude of the signal received by PZT patches was obviously attenuated with the increases of debonding area. Moreover, the signal amplitude of the damage region was quite different from the healthy region. The attenuation of wave in air is much greater than that in concrete. That is the reason why the amplitude was attenuated after the interfacial debonding occurred. Therefore, it can be preliminarily indicated that the detection method can effectively identify the interfacial damage of CFGC. Based on the acquired sweep signal, we calculated the wavelet packet energy of the signal under different debonding area. As shown in Figure 9a, with the increase of the debonding area, the wavelet packet energy decreased greatly. Figure 9b is a comparison of the damage coefficient of A1 based on wavelet packet energy. It can be seen from Figure 9b that the damage index DI was sensitive to the damage of different debonding area. Thus, this method can effectively identify the damage of different debonding area.
(2) Analysis for Delay of Pulse
  In order to further verify the effectiveness of the active detection method based on piezoelectric transducers in the damage identification of interfacial debonding on CFGC. The pulse was applied to excite the SA1 of specimen A1, and the acquired waveform is shown in Figure 10. The arrival time of pulse under four kinds of damage condition was extracted, as shown in Figure 11a. It can be seen from Figure 11a that the arrival time of pulse was delayed with the increases of the damage area. The pulse delay was calculated based on the health state referenced. It can be speculated that the propagation path of waves was forced to change with the occurrence of damage, which may cause a detour propagation of waves. Moreover, the larger the damage area was, the larger wave detour distances, and the delays were more obvious. The regression analysis in Figure 11b showed that when the damage area was less than or equal to 6000 mm^2^, the debonding area was linear with the delay of the pulse wave, and the fitting curve was in good agreement with the test results.

#### 4.2.2. Test of Different Debonding Thickness Identification

For the specimen A2, sweep and pulse signals were also used for excitation respectively. In addition to the wavelet packet energy and damage index DI based on sweep, the delays based on pulse were calculated. The test results were listed in Table 4.

**Figure 12:**
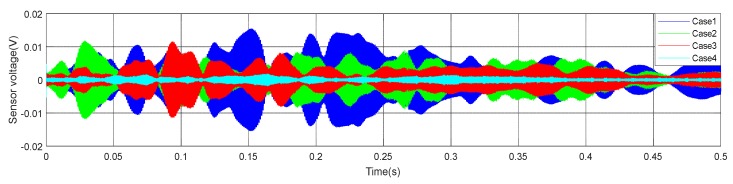
Waveform of specimen A2 under sweep excitation.

**Figure 13:**
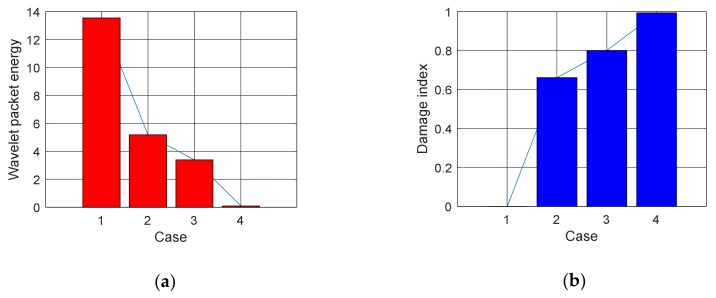
Wavelet packet energy and damage index under four cases of specimen A2. (**a**) Wavelet packet energy; (**b**) Damage index DI.

**Figure 14:**
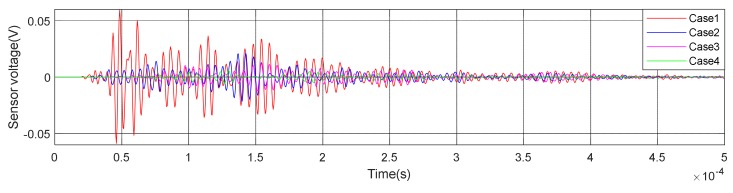
Waveform of specimen A2 under pulse excitation.

**Figure 15:**
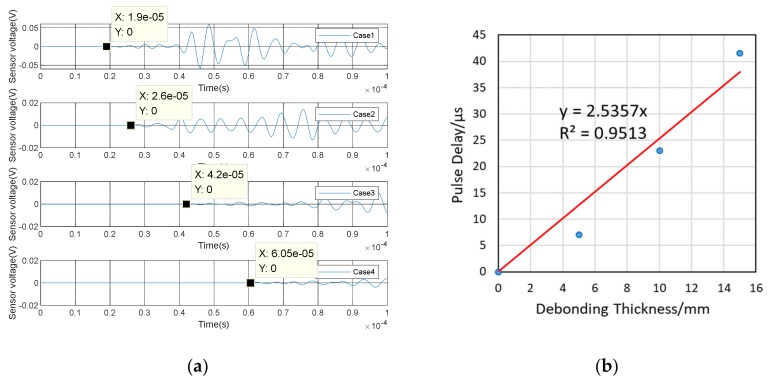
Test results of specimen A2 under pulse excitation. (**a**) Arrival time of pulse wave; (**b**) Relationship between debonding thickness and pulse delay.

(1) Time Domain Analysis of Sweep Signal
  The time domain of signal under the sweep excitation is shown in Figure 12 of specimen A2. It can be seen from the Figure 12 that the amplitude of the signal received by PZT patches was significantly attenuated with the increases of debonding thickness. Moreover, the signal amplitude of the damaged region was very different from the healthy region. It can be preliminarily indicated that the detection method can effectively identify the interfacial damage with different debonding thicknesses of CFGC. Figure 13a was a comparison for the wavelet packet energy under different damage thicknesses. It can be seen from Figure 13a and Table 4 that the energy value was decreasing with the transformation of the interface condition of CFGC from health to damage. Compared with healthy status, when the debonding thicknesses were 5 mm and 10 mm, the wavelet packet energy was respectively decreased by 61.7% and 75.0%. When the debonding thickness was 15 mm, the energy of signal was greatly attenuated, and the value almost closed to 0, which can prove that the wavelet packet energy was sensitive to the change of debonding thickness. The DI based on wavelet packet energy was also calculated, as shown in Figure 13b. Combined with Table 4, it can be seen that the damage index DI was sensitive to the change of debonding thickness, and DI can better characterize the damage level of specimens with different debonding thicknesses.
(2) Analysis for Delay of Pulse
  In order to further verify the sensitivity of the detection method in the different debonding thicknesses of CFGC, the pulse was used to excite the SA2, and the acquired waveform was shown in Figure 14. The arrival times of pulse under four kinds of damage conditions were extracted, as shown in Figure 15a. It can be seen from the Figure 15b that the arrival time of pulse was delayed with the increase of damage thickness. It can be speculated that the wave propagation path was forced to change with the occurrence of damage, which caused a detour of wave. Further, the bigger the damage thickness was, the more the obvious delay. The delay of pulse wave was calculated based on case 1. As shown in Figure 15b, we performed a regression analysis on the pulse delay - debonding thickness relationship, which is a straight line, and the fitting curve was in good agreement with the test results. Therefore, we can speculate that when the debonding thickness is 15 mm or less, there is a linear relationship between the pulse delay and the debonding thickness.

## 5. Discussion

In this study, we got that there is a linear relationship between the pulse delay and the debonding area (or the debonding thickness), and these correlations are true in this paper. However, this test used small samples, small specimens, and the artificial damage, and a connection of scaling up was not done in this study, so, there is no guarantee how the linear relation will hold up in larger samples. At present, our research in this area is still in the gradual and in-depth exploration stage. In future, we will do more experiments based on this research to prove the applicability of these correlations in more samples.

## 6. Conclusions

In this paper, the SAs embedded and the PZT patches bonded were combined to monitor and analyze the interfacial debonding damage of CFGC, and an active monitoring method was adopted. The test results showed that:

(1) The monitoring method can effectively identify the debonding damage region of CFGC, and the damage index proposed in the paper was sensitive to the damage level.
(2) The test results showed that both sweep excitation and pulse excitation can effectively identify the debonding regions of CFGC with different area and thicknesses. In addition to the wavelet packet energy based on sweep, the arrival time based on pulse was sensitive to the damage degree.
(3) It was found that there was a linear relationship between the debonding area (or thickness) and the delay of pulse by regression analysis. Therefore, it can be inferred that the occurrence of damage caused the pulse detour during the process of wave propagation, and the delay of pulse was more obvious with the increase of damage.

The monitoring method proposed in this paper adopts one-excitation with multiple receptions. Further, it is simple in operation, high in flexibility, and its economic benefits are obvious. Therefore, it is an effective method for evaluating interfacial debonding damage of CFGC and it has a broad application prospect. In practical engineering applications, PZT patches can be installed on the tentacles of a crawling robot, and the interfacial cementation of CFGC can be detected and analyzed by this method, which can replace traditional detection methods to realize artificial intelligence automatic detection. However, this paper is a confirmatory empirical research, that is, an exploratory study for whether the active sensing method based on PZT transducer can effectively identify the interface damage of CFGC. Moreover, this test used small samples, small specimens, and the damage was created artificially. Hence, there is still a certain gap between this test and actual engineering. In fact, this test is just a foundation. If the research results were to be applied to practical engineering, a full-scale test would need to be carried on specific components in the structure, which will require a further study.

## Figures and Tables

**Table 1:** Test parameters of specimen A1.

Incentive Method and Parameters			Acquisition Parameter		Filtering Parameter	
Sweep	Amplitude	10 V	Frequency	1 MHz	Filter	Butterworth
	Range	50 kHz–350 kHz	Length	512 k	Type	Bandpass
	Frequency	0.6 kHz	Sampling time	524 ms	Order	5
	Step size	1 ms	Trigger mode	Normal	Range	30 kHz–350 kHz
Pulse	Amplitude	100 V	Frequency	2 MHz	Filter	Butterworth
	Width	2 μs	Length	1 k	Type	Bandpass
	Number of coded bits	20	Sampling time	512 ms	Order	5
	Duration	40 μs	Trigger mode	Normal	Range	150 kHz–500 kHz

**Table 2:** Test parameters of specimen A2.

Incentive Method and Parameters			Acquisition Parameter		Filtering Parameter	
Sweep	Sweep amplitude	10 V	frequency	1 MHz	Filter	Butterworth
	Sweep range	30 kHz–230 kHz	length	512 k	Type	Bandpass
	Step frequency	0.4 kHz	Sampling time	524 ms	Order	5
	Step size	1 ms	Trigger mode	Normal	Range	10 kHz–230 kHz
Pulse	amplitude	100 V	frequency	2 MHz	Filter	Butterworth
	Width	2 μs	length	1 k	Type	Bandpass
	Number of coded bits	20	Sampling time	512 ms	Order	5
	Duration	4 μs	Trigger mode	Normal	Range	150 kHz–400 kHz

**Table 3:** Experimental results of specimen A1.

Specimen A1	Debonding Area	Wavelet Packet Energy Value/*V^2^*	Damage Index *DI*	The Arrival Time of Pulse/*μs*	Delay of Pulse/*μs*
Case 1	0 mm^2^	57.7876	0	21.5	0
Case 2	2000 mm^2^	16.5961	0.6965	36.0	14.5
Case 3	4000 mm^2^	12.8845	0.7809	44.5	23.0
Case 4	6000 mm^2^	5.0520	0.9084	57.5	36.0

**Table 4:** Experimental results of specimen A2.

Specimen A2	Debonding Thickness	Wavelet Packet Energy Value/*V^2^*	Damage Index *DI*	The Arrival Time of Pulse/*μs*	Delay of Pulse/*μs*
Case 1	0 mm	13.5495	0	19	0
Case 2	5 mm	5.1830	0.6608	26	7
Case 3	10 mm	3.3962	0.7997	42	23
Case 4	15 mm	0.0983	0.9931	60.5	41.5
